# *Agastache* honey has superior antifungal activity in comparison with important commercial honeys

**DOI:** 10.1038/s41598-019-54679-w

**Published:** 2019-12-03

**Authors:** Sushil Anand, Margaret Deighton, George Livanos, Edwin Chi Kyong Pang, Nitin Mantri

**Affiliations:** 1 The Pangenomics Group, School of Science, RMIT University, Melbourne, 3083 Victoria Australia; 2 Kenkay Pharmaceuticals Pty Ltd., Smeaton Grange, 2567 NSW Australia

**Keywords:** Antifungal agents, Fungi

## Abstract

There is an urgent need for new effective antifungal agents suitable for the treatment of superficial skin infections, since acquired resistance of fungi to currently available agents is increasing. The antifungal activity of mono-floral *Agastache* honey and commercially available honeys were tested against dermatophytes (*T. mentagrophytes* and *T. rubrum)* and *C. albicans* (ATCC 10231 and a clinical isolate) by agar well diffusion and micro-dilution (AWD and MD). In AWD and MD assays, *Agastache* honey was effective at 40% concentration against dermatophytes (zone diameter, 19.5–20 mm) and *C. albicans* with the same MIC and MFC values indicating fungicidal activity. Tea tree honey was effective at 80% concentration (zone diameter, 14 mm) against dermatophytes and at 40% concentration against *T. mentagrophytes* and *C. albicans*. Manuka was effective at 80% concentration only against *T. mentagrophytes* (zone diameter, 12 mm) and at 40% against *T. rubrum* and *C. albicans* with fungistatic activity. Similar to the AWD results, Jelly bush, Super Manuka, and Jarrah showed no activity against dermatophytes but showed some activity against *C. albicans*. Headspace volatiles of six honeys were isolated by SPME and identified by GC-MS. The characteristic chemical markers for each honey were as follows: *Agastache*- Phenol, 2,4-bis(1,1-dimethylethyl) and Estragole; Manuka and Tea-tree- Acetanisole and Methyl 3,5-dimethoxybenzoate; Jelly bush- Linalool and Nonanal; Super Manuka- Methyl 3,5-dimethoxybenzoate and Nonanal; Jarrah- Isophorone and Nonanoic acid. Overall, analysis of the bioactive compound content and antifungal activity of *Agastache* honey indicated possible use as an antifungal agent for management of superficial fungal infections.

## Introduction

The health benefits of honey have been known for centuries and have recently gained acceptance in the modern medicine. Recently, honey has been reported to possess many biological properties including antimicrobial, antibiofilm, antioxidant, anti-inflammatory and wound healing activities^1–4^. Furthermore, medical-grade honey has been used in wound dressing with the successful reduction of wound size^5^. An increase in resistance to currently available antifungal agents for skin-infections caused by *C. albicans* and the dermatophytes has also been reported^6,7^. Hence, development of new antifungal agents is important.

Honey is a viscous functional food which comprises 81% sugar, 17% water and 2% of other compounds. These compounds include non-volatile products such as enzymes, phenolic compounds and flavonoids as well as volatile compounds, all of which influence the pharmacological properties of honey. The antimicrobial activity of honey is chiefly due to hydrogen peroxide, the osmotic effect, pH and various phenolic compounds^8,9^. Larsen and White, (1995) reported that all 38 clinical isolates of *Candida* spp. examined were inhibited by hydrogen peroxide at pH 4 and pH 7 confirming the role of hydrogen peroxide^10^. Similar results were obtained by Carter *et al*.^11^. However, in addition to hydrogen peroxide, chemical compounds (volatile and semi-volatile) in honey play significant role in exerting biological activity^12^.

Volatile organic compounds of honey, responsible for its flavour and aroma, are mainly sourced from plant nectar. The volatile compound profile of any honey is specific to the plant species and geographical origin of the honey^12^. More than 600 volatile compounds have been isolated from honey. These compounds originate from different biosynthetic pathways and belong to different chemical families^13^. The composition and concentration of these compounds may vary to some extent between batches due to seasonal variation, even if the honey is sourced from a single flower (mono-floral honey). However, the composition of volatile compounds of mono-floral honeys is generally specific and characteristic of honey type whereas the composition of poly-floral honeys is more variable.

Honey contains the three categories of volatile compounds: terpenes, norisoprenoids and benzene derivatives^14^. Terpenes contain a chain of isoprene and can be categorized as monoterpenes, sesquiterpenes, diterpenes, triterpenes or tetraterpenes. The most common monoterpenes identified are linalool and its derivatives, β-terpineol, dihydrocitronellol, β-citronellol, citronellal, geranyl acetone, limonene, β-pinene, tetrahydrogeraniol, cavacrol, p-cymene, 1,8-cineol, camphor, isoborneol and p-cymenol^12,15,16^. Most of these compounds show activity against bacteria, viruses and fungi^17^. Norisoprenoids include α-isophorone, β-isophorone, β-damascenone and 4-oxoisophorone^18^. These compounds have radical scavenging capacity^19^. Benzene derivatives such as benzene acetaldehyde, benzaldehyde, and benzene ethanol are also present in honey^20^. Benzene derivatives identified in New Zealand Manuka and Kanuka honey include methyl 4, hydroxy-3, 5-dimethoxy benzoate and methyl 3, 4, 5-trimethoxy benzoate^21^.

The method used to extract volatiles can also determine the range of compounds identified. Methods of extraction include ultrasonic solvent extraction (USE), hydro-distillation (HD), liquid-liquid extraction (LLE), simultaneous steam distillation extraction (SDE), and solid phase extraction (SPE) with the help of solvents and heat. To eliminate the effects of toxic solvents, the preferred method of extraction is head space (HS) Solid Phase Micro-extraction (HS-SPME)^22^. The benefits of HS-SPME compared to other methods include short sample preparation time and ability to quantify a large number of molecules in a short space of time. Moreover, the fibres used for the extraction of volatiles are selective^23^. This method has been utilized to nominate specific chemical markers in honey from various parts of the globe. Some of the reported markers for honey are listed in Table 1.

**Table 1:** Chemical markers assigned to honeys.

Method Employed	Honey type	Floral Marker	Reference
HS-SPME GC-MS	Acacia honey	Cis-linalool oxide and heptanal	^70^
HS-SPME GC-MS	Chestnut honey	2-aminoacetophenone, 1-phenylethanol	^71^
HS-SPME GC-MS	Lime-tree honey	Carvacrol and *p*-cymene	^72^
HS-SPME GC-MS	Eucalyptus honey	2-hydroxy-5-methyl-3-hexanone, 3-hydroxy-5-methyl-2-hexanone	^62^
HS-SPME GC-MS	Citrus honey	limonyl alcohol, sinensal isomers, and α-4-dimethyl-3-cyclohexene-1-acetaldehyde	^63^
HS-SPME GC-MS	Ulmo honey	4-vinylanisole, benzylaldehyde, ethyl benzoate, ethyl anisate, lyrame, linalool and damascenone	^56^

Honeys with potent antibacterial activity may not necessarily have antifungal activity, for instance; Manuka honey has good antibacterial activity but weak activity against *C. albicans* and dermatophytes^24^. Therefore, it becomes necessary to test the efficacy of honeys known to have antimicrobial activity against common dermatophytes and *C. albicans*. Recently, we produced mono-floral honey from *Agastache rugosa (Lamiaceae family)* grown under controlled conditions and characterised its physicochemical properties and antioxidant capacities, antimicrobial activity and bioactive compounds^3,25^. *A. rugosa (Lamiaceae family)*, a medicinal plant is commonly known as Korean Mint, is widely grown in the fields of Korea, China, and Japan. In traditional Chinese medicine, its leaves have been used to treat cholera, vomiting and miasma and have been reported to have antimicrobial, antifungal, antitumor and cytotoxic activities^26–28^. In addition to its use in traditional medicines, it is used as wild vegetable and cultivated commercially for use as a flavouring agent^29^. Hence, we hypothesize that at least some of the products derived from this plant have antifungal activity.

The aims of the present study were to (i) assess the antifungal activity of *Agastache* honey and important commercial honeys derived from *Leptospermum* species (New-Zealand Manuka honey, Australian Tea tree honey, Jelly bush honey, and Super manuka honey) and Australian Jarrah honey, (ii) Characterize the volatile compounds present in *Agastache* honey and flowers (containing nectar) (iii) Compare the volatile compound profile of *Agastache* honey with that of important commercial honeys (iv) Identify compounds in *Agastache* honey and other honeys with possible antifungal activity.

## Results

### Agar well diffusion assay (*T. mentagrophytes* and *T. rubrum*)

**Figure 1:**
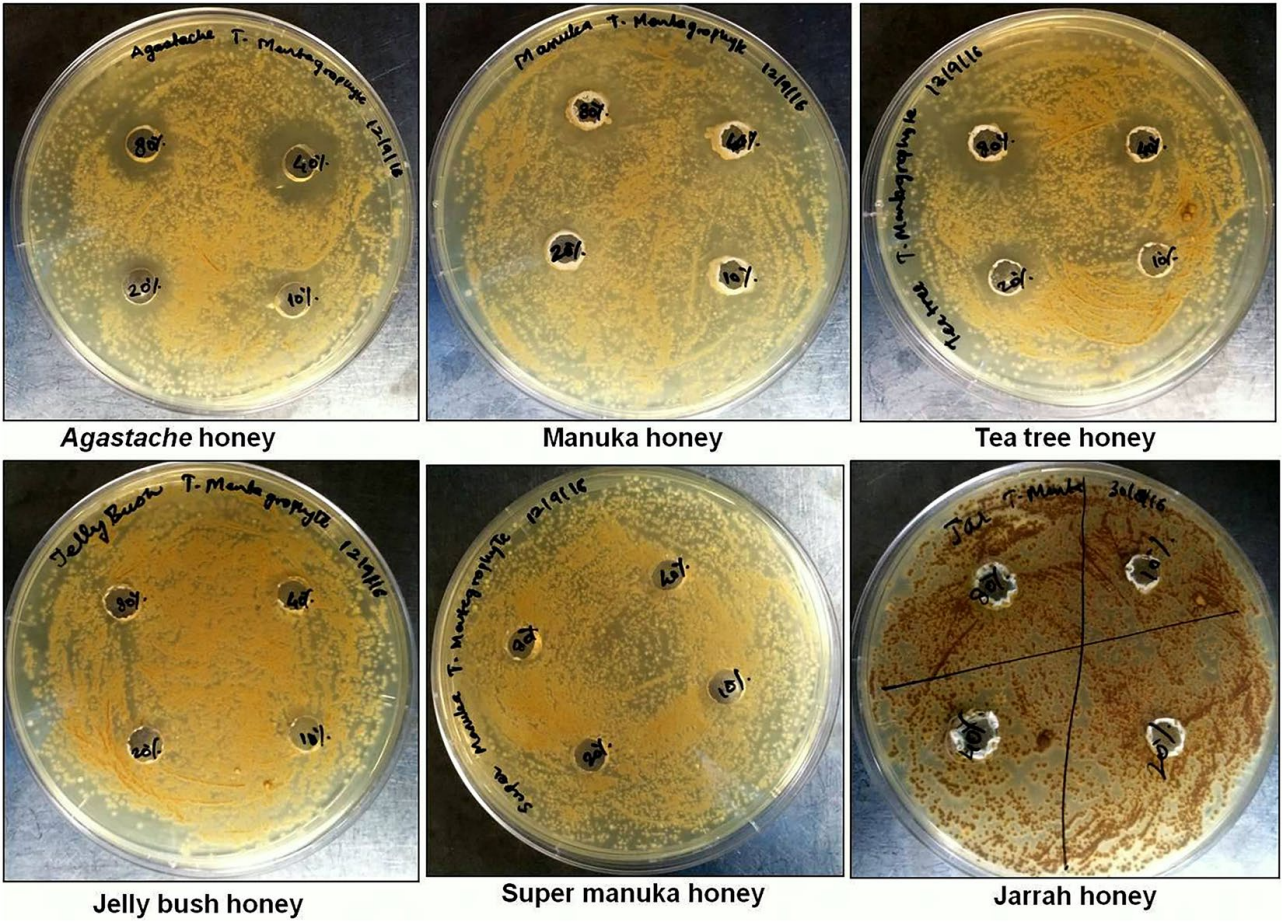
Antifungal activity of honey against *T. mentagrophytes* determined by agar well diffusion. Honeys were tested in the range of 80–10% (w/v). *Agastache* honey exhibited the largest zone of inhibition at 40% concentration (20 mm) followed by tea-tree honey and Manuka honey at 80% concentration (14 mm and 12 mm, respectively).

**Figure 2:**
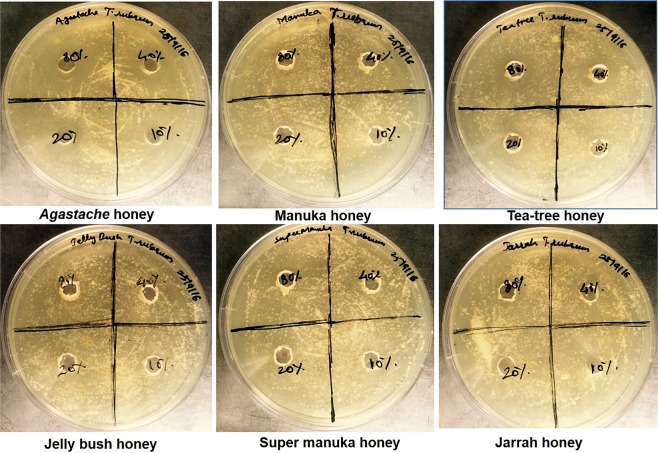
Antifungal activity of honey against *T. rubrum* determined by agar well diffusion. Honeys were tested in the range of 80–10% (w/v). *Agastache* honey exhibited the largest zone of inhibition at 40% concentration (19.5 mm) followed by tea-tree honey at 80% honey (14 mm).

The antifungal activity of *Agastache* honey, Leptospermum-origin honeys and Jarrah honey was tested against *T. mentagrophytes* and *T. rubrum* by the well-diffusion assay. Table 2 shows the inhibition zone sizes produced by honey samples tested at different concentrations (80–10%). *Agastache* honey exhibited strong antifungal activity against both the isolates. The zone of inhibition of honey at various concentrations for *Agastache* honey against *T. mentagrophytes* ranged from 20 mm to 10 mm and against *T. rubrum* ranged from 19.5 to 12 mm. Manuka exhibited weak activity against *T. mentagrophytes* but showed no activity against *T. rubrum*. Tea tree honey exhibited moderate activity against both isolates with zones ranging from 11–14 mm against *T. mentagrophytes* and 12–14 mm against *T. rubrum*. Jelly bush, Super Manuka and Jarrah honey showed no activity against either isolate. The images depicting the zone of inhibition for honeys against both isolates are given below in Figs. 1 and 2.

**Table 2:** Antifungal activity of honey at different concentrations, hydrogen peroxide content and major volatile compounds of honeys.

Honey type	Honey source (species)	Agar well diffusion (zone size in mm)								Micro-dilution (MIC, % honey)				Hydrogen peroxide	Major volatile compounds
		*Trichophyton mentagrophytes*				*Trichophyton rubrum*				*Trichophyton mentagrophytes*	*Trichophyton rubrum*	*Candida albicans* ATCC-10231	*Candida albicans* clinical isolate	(µM) at 40% honey conc.	
		80%	40%	20%	10%	80%	40%	20%	10%						
*Agastache*	*Agastache Rugosa*	15 ± 0.5	20 ± 0.5	10 ± 0.3	0	16 ± 0.3	19.5 ± 0.4	12 ± 0.3	0	40	40	40	40	5.13 ± 2	Phenol, 2,4-bis(1,1-dimethylethyl), Estragole and Nonanoic acid, ethyl ester
Manuka	*Leptospermum scoparium*	12 ± 0.7	0	0	0	0	0	0	0	0	40	40	40	0.04 ± 0.5	Acetanisole, and Methyl 3,5-dimethoxybenzoate
Tea Tree	*Leptospermum lanigerum* & *Leptospermum scoparium*	14 ± 0.5	12 ± 0.3	11 ± 0	0	14 ± 0.3	12 ± 0.4	0	0	40	0	40	40	155 ± 5.7	Acetanisole, and Methyl 3,5-dimethoxybenzoate
Jelly Bush	*Leptospermum polygalifolium*	0	0	0	0	0	0	0	0	0	0	40	0	13.02 ± 0.5	Linalool and Nonanal
Super Manuka	*Leptospermum polygalifolium*	0	0	0	0	0	0	0	0	0	0	0	0	41.30 ± 0.5	Methyl 3,5-dimethoxybenzoate and Nonanal
Jarrah	*Eucalyptus marginata*	0	0	0	0	0	0	0	0	0	0	40	0	115 ± 9.1	Isophorone and Nonanoic acid

The antifungal activity of honey was assessed against dermatophytes and *C. albicans*. The hydrogen peroxide content of honeys was determined at a concentration of 40%. Data represent the mean of triplicate readings ± standard deviations (SD).

### Broth micro-dilution assay

**Figure 3:**
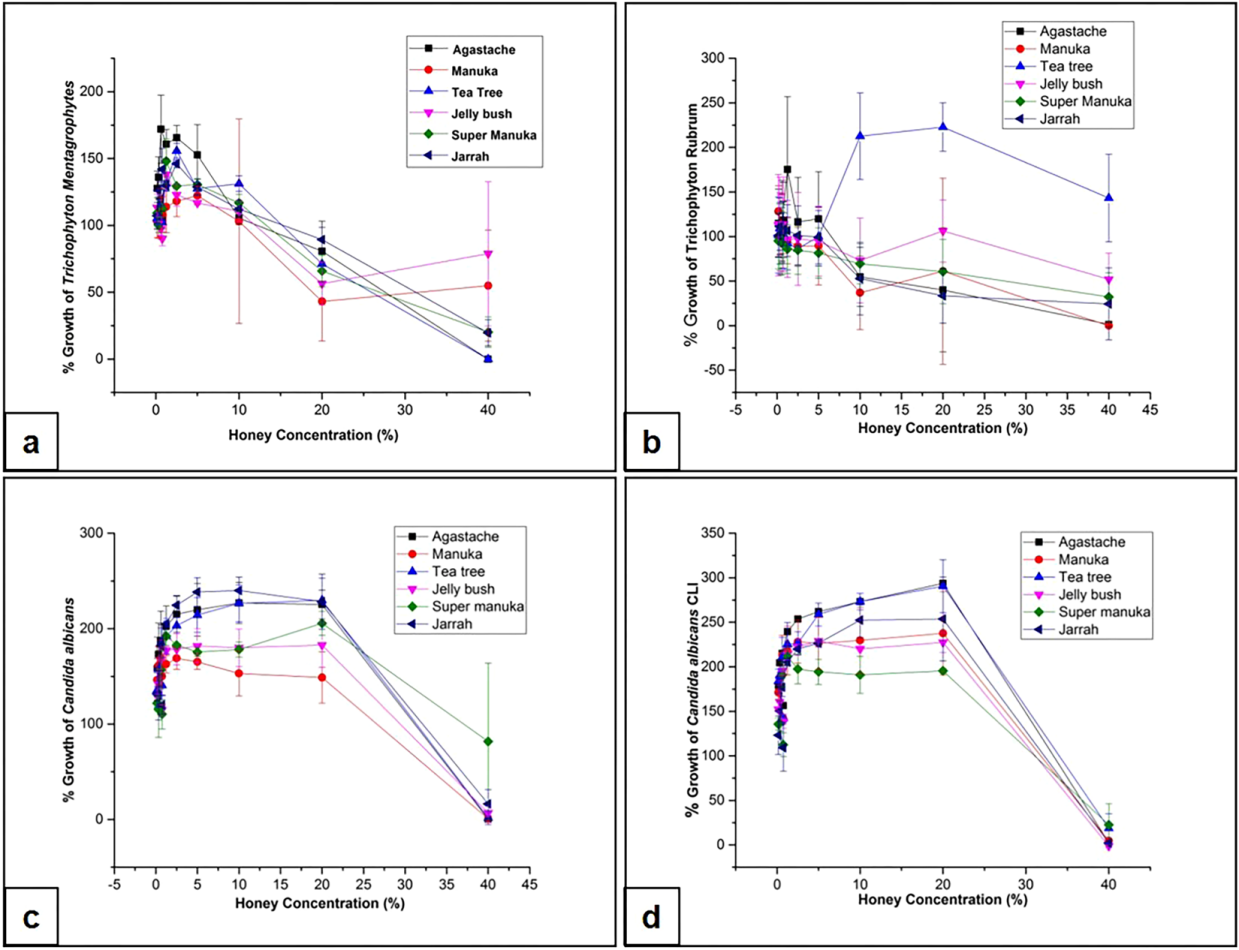
Antifungal activity of honeys measured by broth dilution. The graph shows the amount of growth (%) after exposure to honey at concentrations ranging from 0% to 40%. *Agastache* (square, black); Manuka (circle, red); Tea-tree (upwards triangle, blue); Jelly bush (downwards triangle, pink); Super Manuka (diamond, green); Jarrah (left triangle, navy) against *T. mentagrophytes* (**a**), *T. rubrum* (**b**), *C. albicans* ATCC 10231 (**c**) and a clinical isolate of *C. albicans* (**d**). Each point represents the mean of triplicate readings ± standard deviations (SD).

All honeys showed some antifungal activity in the broth dilution assay. The activity of six honeys against four fungal strains including clinical isolates varied, depending on the species and strain and the type of honey (Fig. 3a–d). In general, all honeys were more effective against *C. albicans* than against the two dermatophytes. The dermatophytes grew in the presence of lower concentrations of all honeys, as did *T. rubrum* in the presence of tea tree honey. However, at 40% honey concentration, all strains with the exception of *T. rubrum*/tea tree honey, showed some inhibition. *Agastache* honey was consistent in inhibiting growth of dermatophytes and yeasts at this honey concentration. The MIC and MFC values were the same for all the strains/isolates, indicating the fungicidal activity of honey. Among *Leptospermum* origin honeys, Manuka honey (40%) was effective against *T. rubrum* and *C. albicans* but only partially effective (45% inhibition) against *T. mentagrophytes*. Manuka honey showed only fungistatic activity. Tea tree honey (40%) inhibited the growth of *T. mentagrophytes* and *C. albicans* and exhibited fungicidal activity but was partially (50% inhibition) effective against *T. rubrum* and *C. albicans* clinical isolates. Other honeys such as Jelly bush (<50% inhibition), Super Manuka (60–70% inhibition), and Jarrah (75–80% inhibition) showed variable activity against dermatophytes whilst, Jelly bush and Jarrah showed only fungistatic against *C. albicans* clinical isolate.

Statistical analysis showed that interaction between three parameters, honey concentration, honey type and fungal strains were significant and affected the growth of all fungal isolates/strains and the *C. albicans* reference strain (ATCC 10231). The main influence on activity was honey concentration, which explained 33% of the variation, fungal strain/isolate (25% of the variation), while honey concentration and fungal strain/isolate interaction explained 16% of variation (Table 3). The non-parametric Kruskal-Wallis test also showed that honey concentration had a significant effect on fungal growth (*p* = 0.001).

**Table 3:** Analysis of variance (ANOVA) main effect of independent variables: tests of independent variables.

Source	DF	Adj SS	Adj MS	F-Value	P-Value
Honey Concentration	10	1261350	126135	154.60	0.001
Honey Type	6	78347	15669	19.21	0.001
Fungal Strain	4	981169	327056	400.86	0.001
Honey Concentration *Honey Type	60	139183	2784	3.41	0.001
Honey Concentration *Fungal Strain	40	611643	20388	24.99	0.001
Honey Type*Fungal strain	24	53907	3594	4.40	0.001
Honey Concentration*Honey Type*Fungal strain	240	224330	1496	1.83	0.001
Error	528	430784	816		
Total	912	3780713			
S	R-sq	R-sq(adj)	R- sq(pred)		
28.56	88.61%	82.93%	74.36%		

### Hydrogen peroxide concentrations in honeys

**Figure 4:**
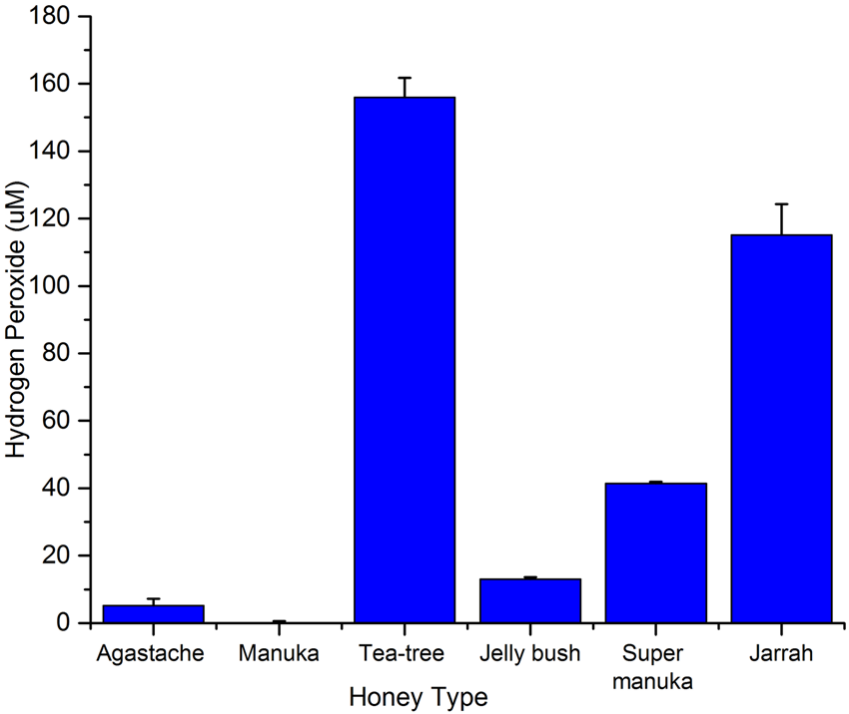
Effect of honey dilutions (40%) on the production of hydrogen peroxide (µM). Data represent the mean of triplicate readings ± standard deviations (SD).

H_2_O_2_ concentrations ranged from 155 µM to 0.04 µM in the different honeys (Fig. 4). At 40% concentration tea tree honey produced the highest amount of hydrogen peroxide (155 µm), Jarrah and Super Manuka honey produced 115 µm and 41 µm of H_2_O_2_ whereas Manuka (0.04 µm), Jelly bush (13 µm) and *Agastache* (5.13 µm) produced low amounts of hydrogen peroxide.

### Head space-solid phase microextraction gas chromatography-mass spectroscopy (HS-SPME GC-MS)

The volatile compounds in six honey samples were isolated by SPME and identified by GC-MS. Samples were analysed in three groups namely *Agastache* honey and *Agastache* flower, honeys derived from *Leptospermum* origin, and Jarrah honey. As replicates differed in the number of volatile compounds detected in each replicate, all three replicates were analysed. The number of common compounds found in all three replicates were; 32 in *Agastache*, 46 in Manuka, 26 in Tea-tree, 38 in Jelly bush, 38 in Super Manuka, 43 in Jarrah honey **(**Supplementary Tables S1–S6**)**.

### Volatile compounds in agastache honey

**Figure 5:**
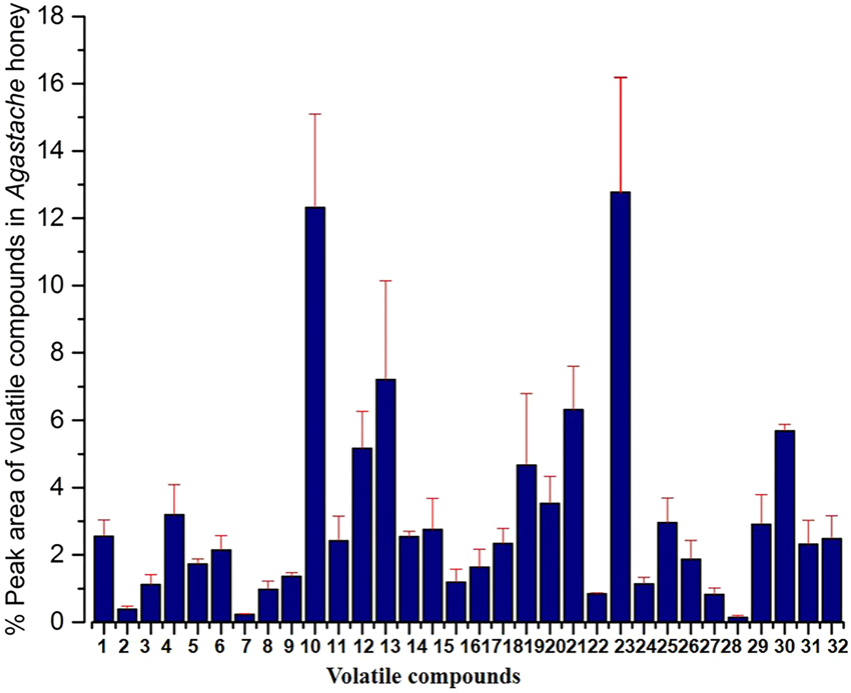
HS-SPME GC-MS volatile compounds identified in *Agastache* honey. Compounds were identified with GC-MS reference libraries (Adams, Wiley 7th and NIST 2.0) using a 70% similarity match cut-off value. The peak area in the total ion chromatograms was the basis of calculations of concentration of studied compounds. The error bar represents the mean of triplicate readings ± standard deviations (SD). 1-Benzaldehyde, 2-D-Limonene, 3-Benzeneacetaldehyde, 4-Nonanal, 5-Phenylethyl Alcohol, 6-1H-Pyrazole, 4,5-dihydro-5,5-dimethyl-4-isopropylidene-, 7-Cyclopentasiloxane, decamethyl-, 8-4,Ketoisophorone, 9-Octanoic acid, ethyl ester, 10-Estragole, 11-Decanal, 12-Benzaldehyde, 4-methoxy, 13-Nonanoic acid, ethyl ester, 14-Benzene, 1-methoxy-4-propyl, 15-Phenol, 2,3,5-trimethyl-, 16-Nonanoic acid, 17-Propanoic acid, 2-methyl-, 2-ethyl-3-hydroxyhexyl ester, 18-2H-Benzimidazol-2-one, 1,3-dihydro-5-methyl-, 19-β-Caryophyllene, 20-Benzoic acid, 4-methoxy-, ethyl ester, 21-2-Propenoic acid, 3-phenyl-, ethyl ester, 22-Y-cadinene, 23-Phenol, 2,4-bis(1,1-dimethylethyl), 24-Benzoic acid, 3,5-dimethoxy-, methyl ester, 25-Dodecanoic acid, ethyl ester, 26-Y-Eudesmol, 27-Heptadecane, 28-Homosalate, 29-Nonadecane, 30-Hexadecanoic acid, ethyl ester, 31-Heneicosane, 32-9,12-Octadecadienoic acid (Z,Z). Data represent the mean of triplicate readings ± standard deviations (SD).

The important volatile compounds in *Agastache* honey were (% peak area): Phenol, 2,4-bis(1,1-dimethylethyl) (12.77%), Estragole (12.31%), Nonanoic acid, ethyl ester (7.22%), 2-Propenoic acid, 3-phenyl-, ethyl ester (6.32%), Hexadecanoic acid, ethyl ester (5.68%), Benzaldehyde, 4 methoxy (5.17%), β-Caryophyllene (4.67%), Nonanal (3.19%), and 2H-Benzimidazol-2-one, 1,3-dihydro-5-methyl- (2.34%). All identified volatile compounds are shown in the Fig. 5.

### Volatile compounds derived from honey of *Leptospermum* origin

**Figure 6:**
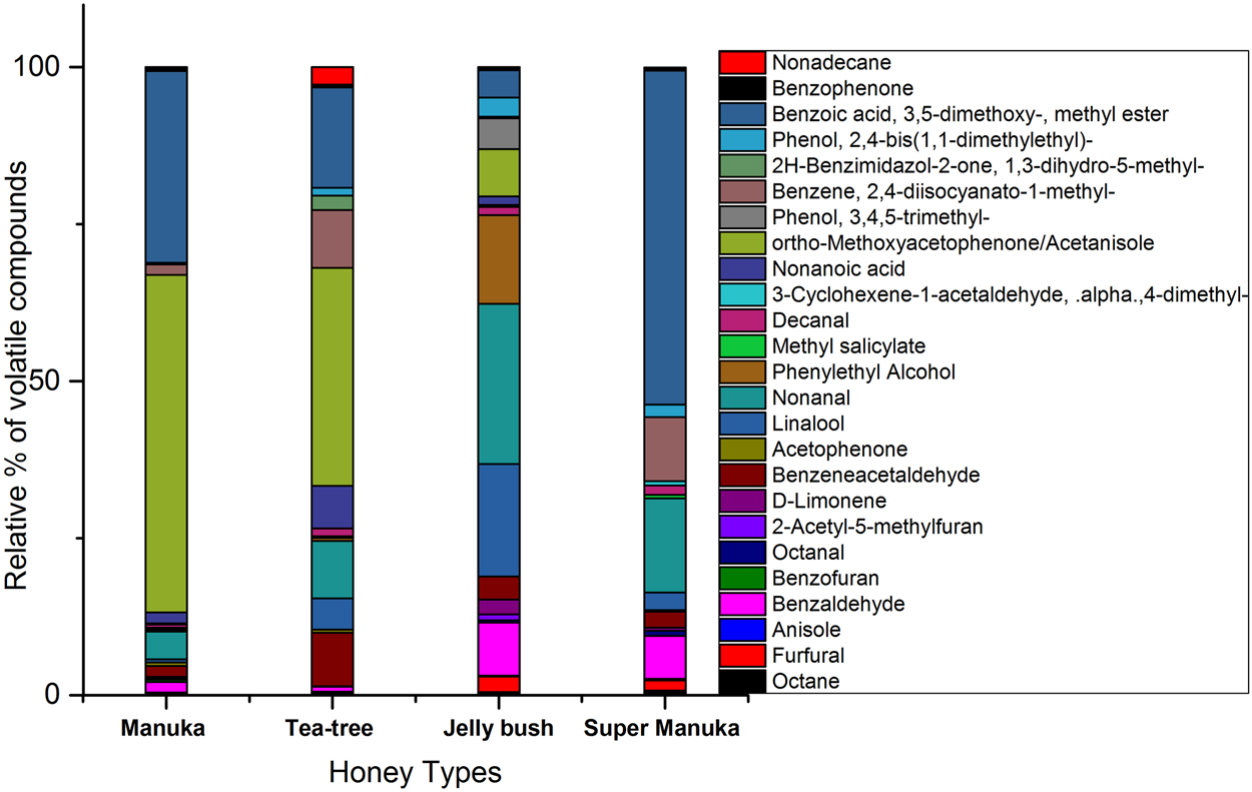
Detection of common volatile compounds by HS-SPME GC-MS in *Leptospernum*-origin honeys (Manuka, Tea-tree, Jelly bush and Super Manuka). Compounds were identified with GC-MS reference libraries (Adams, Wiley 7th and NIST 2.0) using a 70% similarity match cut-off value. The peak area in the total ion chromatograms was the basis of calculations of concentration of studied compounds. The relative % of common volatile compounds were calculated from the total peak area of the volatile compounds.

Many common compounds were observed in all honeys as shown in Fig. 6. The major volatile compounds detected in each honey were as follows; Manuka contained Acetanisole/ortho-Methoxyacetophenone (39%), Methyl 3,5-dimethoxybenzoate (26.67%), Anisole (4.34%), Nonanal (3.87%) and Ethanone, 1-(2-hydroxyphenyl) (1.29%), Tea-tree contained Acetanisole (31.3%), Methyl 3,5-dimethoxybenzoate (14.47%), 2,4-Diisosynato-1-methylbenzene (8.31%), Nonanal (8.25%), Benzene acetaldehyde (7.7%), Nonanoic acid (6.12%), Linalool (4.5%) and Nonanol (4.5%), Jelly bush contained Linalool (19.4%), Nonanal (12.4%), Methyl 3,5-dimethoxybenzoate (11.7%), α-Terpineol (9.16%), 3,4,5-trimethylphenol (7.82%), Benzaldehyde (6.75%), p-Cresol (4.34%), and ortho-Methoxyacetophenone (2.93%), Super Manuka contained Methyl 3,5-dimethoxybenzoate (40%), Nonanal (11.3%), 3,4,5-trimethylphenol (8.63%), 2,4-Diisosynato-1-methylbenzene (7.67%), Benzaldehyde (5.12%), 2,3,5-trimethylphenol (4.52%), and Anethole (2.94%).

Of the 80 different species of *Leptospermum* origin native to Australia, honey was available from the following: Jelly bush, Super Manuka and Tea-tree honeys. Manuka is derived from *Leptospermum* genus found in New Zealand. The common major compounds were benzaldehyde, benzacetaldehyde, Linalool, Nonanal and Methyl 3, 5-dimethoxybenzoate. Acetanisole was the major marker compound found in Manuka and Tea tree, whilst it was detected in low amounts in Jelly bush and not in Super Manuka honey. Methyl 3,5-dimethoxybenzoate was detected in all honeys at variable percentages and can be described in the descending order as follows: Super Manuka > Manuka > Tea tree > Jelly bush. Linalool was the marker compound found in Jelly bush honey and a minor compound in Tea tree honey but was detected in trace amounts in other honeys. Benzene 2, 4-diisocynato-1-methyl was detected in Tea tree and Super manuka honey only. Nonanal was another common compound detected in all honeys at variable percentages, in the descending order: Jelly bush > Super Manuka > Tea tree > Manuka.

### Volatile compounds in Jarrah honey

**Figure 7:**
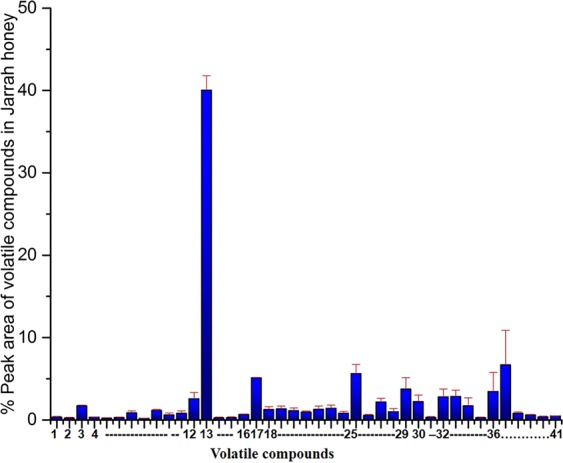
Volatile compounds identified in Jarrah honey by HS-SPME GC-MS. Compounds were identified with GC-MS reference libraries (Adams, Wiley 7th and NIST 2.0) using a 70% similarity match cut-off value. The peak area in the total ion chromatograms was the basis of calculations of concentration of studied compounds. The error bar represents the mean of triplicate readings ± standard deviations (SD). **1**-Methane, thiobis-, **2**-2-Butanone, 3-hydroxy-, **3**-Acetyl valeryl, **4**-(Z)-2-(Aminomethylene)-3,3-dimethylbutanenitrile, **5**-4-Methyl-2-hexanol, **6**-Benzaldehyde, 7-Cymene < Ortho > , **8**-3-Cyclohexen-1-one, 3,5,5-trimethyl-, **9**-Benzeneacetaldehyde, **10**-Cymenene < Para- > , **11**-Linalool, **12**-Nonanal, **13-**Isophorone, **14-**Cyclopentasiloxane, decamethyl-, **15**-Cyclohexanol, 4-(1-methylethyl), **16**-Ethanone, 1-(1,4-dimethyl-3-cyclohexen-1-yl), **17**-2-Hydroxy-3,5,5-Trimethyl-2-Cyclohexenone, **18**-Octanoic Acid, **19**-Terpineol < alpha- > , **20**-Benzenemethanol, alpha., alpha, 4-trimethyl, **21**-1,3-Cyclohexadiene-1-carboxaldehyde, 2,6,6-trimethyl, **22**-Decanal, **23-**Furan, 3-phenyl, **24-**Cumin aldehyde, **25**-Nonanoic acid, **26**-Cymen-7-ol < Para- > **27-**Thymol **28**- Phenol, 2-methyl-5-(1-methylethyl) **29**-Benzene, 2,4-diisocyanato-1-methyl, **30-**Decanoic acid, **31**-2-Propenoic acid, 3-phenyl- **32**-2H-Benzimidazol-2-one, 1,3-dihydro-5-methyl- **33**-9,9-dimethyl-9,-10-dihydroanthacene, **34-**Pentadecane **35-**Coumarin, 3,4-dihydro-4,4,7-trimethyl- **36-**(+−)-(5,6,7,8-Tetrahydro-4-methyl-1-naphthalenyl)-1-ethanone **37-**Benzoic acid, 3,5-dimethoxy-, methyl ester **38-**Benzophenone **39-**2-Cyclohexen-1-one, 3,5,5-trimethyl-4-(3-oxobutyl)- **40-**1,2-Benzenedicarboxylic acid, bis(2-methylpropyl) ester, **41**-Homo menthyl salicylate. Data represent the mean of triplicate readings ± standard deviations (SD).

Jarrah tree (*Eucalyptus marginata)* is native to Western Australian. The major bioactive compounds in Jarrah honey were Isophorone (40.06%), Nonanoic acid (5.62%), 2, hydroxy 3, 5, 5-trimethyl-2 cyclohexenone (5.12%), and 2,4-Diisosynato-1-methylbenzene (3.75%) as shown in Fig. 7. Isophorone is the chemical marker compound which was identified only in this honey. Another major compound was Nonanoic acid which was also found in Jelly bush and Tea tree honey.

## Discussion

The most common dermatophytes causing skin infection in humans are *T. mentagrophytes* and *T. rubrum*^30^. Tinea pedis or athletes foot, tinea cruris or jock itch, *tinea corporus* or ringworm of the body (face, scalp, nail and hand) are the major infections caused by dermatophytes^31^. Candidiasis is most commonly caused by *C. albicans*. Currently, there are many anti-mycotic agents available over the counter and some have become only partially effective^7^. Moreover, some patients prefer to use natural products instead of commercially available antimycotic agents. Therefore, we evaluated honey as an alternative to available antifungal agents. The antifungal activity was assessed by two methods, agar well diffusion (AWD) and micro-dilution (MD).

Based on the AWD assay, *Agastache* honey was the most effective honey against *T. mentagrophytes* and *T. rubrum*, closely followed by Tea tree honey, with Manuka honey showing some activity. The other honeys examined (Jelly bush, Super Manuka, and Jarrah honeys) showed no activity in the AWD assay (Fig. 1, Table 2). The more sensitive MD assay reflected these results, with only *Agastache* honey showing inhibition against both dermatophytes at 40% concentration, while Tea tree honey and Manuka were active against *T. mentagrophytes* and *T. rubrum* respectively. The other honeys examined were less active or showed no anti-dermatophyte activity; indeed *T. rubrum* was able to grow in the presence of Tea tree honey (Table 2). The MIC and MFC values of *Agastache* honey against the two dermatophytes indicated fungicidal activity. Tea tree honey was also fungicidal, but only against *T. mentagrophytes*. No other honeys displayed fungicidal activity against the dermatophytes. The lower sensitivity of the AWD assay compared with MD could be explained by the need for the active components to diffuse across agar; however, AWD is considered to be an appropriate model to assess agents applied topically^32^. Furthermore, the observed zones of inhibition of honey at 80% for *Agastache* honey were smaller than at 40% concentration (Table 2). Generally, the production of hydrogen peroxide increases over the serial dilutions. However, for most of the honeys, accumulation of hydrogen peroxide is maximum at 30–50%^33^. Therefore, the inhibition size variation may be a result of less hydrogen peroxide production at highest concentration (80%).

In general *C. albicans* was more susceptible to all honeys than the dermatophytes (Fig. 3c,d), the only exception being Super Manuka honey that showed no activity against either *Candida* strain (Table 2). *O*nly Tea tree honey showed fungicidal activity against *C. albicans*. The highest concentration of honey used for MIC determination in the present study was 40%. It is possible that honeys such as Jelly bush, Super Manuka and Jarrah might show activity at higher concentration. Notably, *Agastache* honey was effective at 40% concentration against both dermatophytes and both strains of *C. albicans*. The control antifungal, fluconazole failed to inhibit *T. mentagrophytes* in both assays but strongly inhibited *T. rubrum* (21 mm), as well as *C. albicans*, ATCC 10231 and a clinical isolate at a concentration of 128 µg/ml.

The few reports in the literature on antifungal activity of honey against dermatophytes and yeasts partially agree with the findings of the present study. Suhana *et al*.^34^ reported that Manuka honey (UMF 10+) has antifungal activity against *C. albicans* (25% v/v) and dermatophytes (50% v/v), while Brady *et al*.^24^ also reported activity of Manuka honey against *T. mentagrophytes*, although the zone of inhibition (18.4 mm) was wider than found in the current study (12 mm). Koc *et al*.^35^ found that fluconazole-resistant *C. albicans* required 40–80% (MIC values) concentration of Turkish honey (Rhododendron, Orange and Eucalyptus) for inhibition. Another honey, Jujube honey (*Zizyphus spina-christi*) was active against *C. albicans* ATCC 10231 at 40% (w/v) (MIC), with the MFC at 50%^36^. In another study, Carter *et al*.^11^ found that Manuka honey was less effective for treatment of fungal skin infections caused by *C. albicans* and dermatophytes than for bacterial skin infections. This finding was attributed the observation that Manuka honey lacks hydrogen peroxide, which is responsible for the antifungal activities of some honeys^11,24^. However, our results do not support this view since *Agastache* honey, which showed the most antifungal activity according to the assays we used, produced lower amounts of H_2_O_2_ (5.13 µm) than Jarrah and Super Manuka honeys (115 µm and 41 µm respectively), which displayed weak antifungal activity. On the other hand, Tea tree honey produced the highest amount of H_2_O_2_ (155 µM) and showed antifungal activity. These findings clearly indicate that other factors than H_2_O_2_ production, for example, volatile and phenolic compounds, contribute to the antifungal activity. In this study, therefore, we have identified volatile compounds in the honeys and their possible relation to the antifungal activity.

This study is the first to investigate and compare the volatile organic compounds of *Agastache* honey (greenhouse-produced, monofloral) with monofloral tea tree honeys sourced from different regions. The aroma and flavour of honey is influenced by its floral origin as well as honey bee species and processing and storage conditions^37^. Also, this is the first study to characterize volatile compounds in *Agastache* honey and compare the profile with data previously reported from our laboratory on the volatile compounds found in *Agastache* flowers (Yamani *et al*.)^38^. The major compounds identified (Fig. 5) in *Agastache* honey were Phenol, 2,4-bis(1,1-dimethylethyl) (12.77%), Estragole (12.31%), Nonanoic acid, ethyl ester (7.22%), 2-Propenoic acid, 3-phenyl-, ethyl ester (6.32%), Hexadecanoic acid, ethyl ester (5.68%), Benzaldehyde, 4 methoxy (5.17%), β-Caryophyllene (4.67%), Nonanal (3.19%), and 2H-Benzimidazol-2-one, 1,3-dihydro-5-methyl- (2.34%). Limonene was also detected (0.11%), whilst menthone, pulegone, methyl eugenol were detected in trace amounts, although not in all replicates.

In order to investigate the source of the volatile compounds in *Agastache* honey, we compared the findings of the present study with an earlier study from our laboratory^38^ which analyzed greenhouse-grown *Agastache* flowers, leaves and flowers with nectar for the presence of volatile compounds. In that study, the predominant volatile compounds present in were estragole, caryophyllene and D-limonene. Estragole was present in highest percentage in leaves (94.35%), flower (96.74%), and flower with nectar (97.16%). In contrast, our present findings that *Agastache* honey contained only 12.31% estragole suggests that the nectar contained only a low amount of the compound compared to flower and leaves. However, this needs to be further confirmed by analyzing volatile compounds of nectar separately. Jerković and Marijanović^39,40^ reported that the volatile compound composition of nectar, honey-sac and Satsuma Mandarin honey were significantly different. The authors suggested these differences could be due to hive conditions and bee-enzyme activity. Other compounds found in the flower nectar, such as Bicyclo undec-4-ene, 4,11,11-trimethyl-8-methylene- an isomer of caryophyllene (4.67%) and D-limonene (0.38%) were detected in *Agastache* honey confirming that major compounds of nectar were transferred to the honey. Estragole was also detected in Australian Super Manuka honey, but in low amounts (1.92%). Estragole was also identified as the most abundant compound in *A. rugosa* and *A. foeniculum* arial parts (18.6% to over 98%)^41^.

Other major compounds identified only in our *Agastache* honey could be the result of transformation of plant compounds by the metabolism of a bee, microbial and environmental contaminations^12,40^. The *Agastache* honey was produced in the greenhouse to ensure that bees forage onto the single flower species. However, the bees store their food inside the honey-sac, honey could be sourced from other plants before the hive was introduced into the glasshouse. Therefore, some compounds could be introduced from the different plant sources. Moreover, bees interact with plants trichome and carry loads of volatile compounds on the wings and transfer them into the honey. Considering different factors, the above-mentioned compounds were identified in the significant amount in samples, hence they can be nominated as chemical markers of *Agastache* honey.

The volatile compound profile of *Agastache* honey identified many antifungal compounds which could be correlated with the activity demonstrated in the present study. These include estragole, the major volatile component, which was effective against *Trichophyton* species, when used at 1.25 mg/ml concentration in combination with ketoconazole (12.5 µg/ml)^42^. In addition, phenol-2, 4-bis (1, 1-dimethylethyl) (100 µg/ml) has activity against *Aspergillus* indicated by reduction in the germ tube length and presence of an inhibition zone in agar dilution studies^43^. Moreover, 2, 4-di-tert-butylphenol (100 µg/ml) inhibited the growth of fungal cells and biofilm formation of *C. albicans*^44^.

Several studies have also reported that oils containing high concentrations of estragole have antifungal activity. For example, croton zehntneri oil, which contains 84.7% estragole produced significant zones of inhibition against fungi (*C. albicans and B. dermatitidis*)^45^. The essential oil extracted from *Foeniculum vulgare*, which contains a major fraction of estragole, demonstrated antifungal activity against *C. albicans*^46^. In addition, other components identified in *Agastache* honey in the present study were reported to have antifungal activity. For example, nonanoic acid, was identified as the antifungal compound in the roots of *Hibiscus syriacus* which has been used successfully to treat tinea pedis^47^. Moreover, 2-propenoic acid, 3-phenyl, ethyl ester (ethyl cinnamate) were effective against *A. niger* and *C. albicans* than *E. coli* and *S. aureus*^48^. Finally, ethyl cinnamate is also active against *C. albicans*^49^.

The study conducted by Kim J. H. reported that several benzaldehydes (such as 2-hydroxy-3-methoxybenzaldehyde, 2-hydroxy-5-methoxybenzaldehyde) have antifungal activity and structure-activity analysis revealed the presence of hydroxyl group increases the antifungal activity^50^. A promising class of bioactive heterocyclic compounds that exhibit a range of biological activities is benzimidazole. Khabnadideh S. studied the derivatives of benzimidazole (such as 1-Nonyl-1 H-benzo[d]imidazole and 1-Decyl-1 H-benzo[d]imidazole) and evaluated the antifungal activity against *C. albicans* and dermatophytes^51^.

As expected, there were differences in the major volatile compounds identified in the different honeys of *Leptospernum* origin (Table 2, Fig. 6). In agreement with other reports^52–54^ the major compound identified in Manuka honey was Acetanisole (O-Methoxyacetophenone). This compound was also observed in highest amount in Tea-tree honey (31.3%) but was present in only low amounts in Jelly bush honey and was absent in Super Manuka honey. These findings are consistent with the *Leptospermum* species origin of the various honeys; since Manuka honey and Tea-tree honey are derived or partially derived from *Leptospermum scoparium*, while Jelly bush and Super Manuka honeys are derived from *Leptospermum polygalifolium*.

In the present study, methyl 3,5-dimethoxybenzoate was identified for the first time in all *Leptospermum-*origin honeys; however, Kirkpatrick *et al*.^55^ detected methyl syringate as the characteristic antioxidant in *Leptospermum-*origin honeys, using GC-MS and HPLC, Methyl 3,5-dimethoxybenzoate could be formed due to a loss of an oxygen atom from methyl syringate. The percentages of this compound among total volatiles in the different honeys was as follows: Manuka (26.67%), Tea tree (14.47%), Jelly bush (11.7%) and Super Manuka (40%). Since Methyl 3,5-dimethoxybenzoate constituted the highest percentage of volatile compounds of Super Manuka honey it could be nominated as a chemical marker of the honey.

Nonanal was identified in all *Leptospermum-*origin honeys and has not been reported previously in Manuka honey. The concentrations of nonanal in the different honeys of *Leptospermum-*origin were as follows; Manuka (3.87%), Tea tree (8.25%), Jelly bush (12.4%) and Super Manuka (11.3%). It was reported to be present in Chilean Ulmo honey (7.97%)^56^. Also, as found in the present study, Jelly bush was reported to contain Cis-linalool and Nonanal^52^, which were nominated chemical markers for Jelly bush.

Several authors have reported on the anti-*Candida albicans* and antibacterial activity of compounds of *Leptospermum* origin that were identified in this study. These include Methyl 3,5-dimethoxybenzoate (Benzoic acid, 3,5-dimethoxy, methyl ester), which has activity against *Candida albicans*^57^. Linalool, the major component (54.4%) of the essential oil of *Ocimum basilucum*, had antimicrobial effects on eight bacteria and three fungi tested, giving inhibition zones ranging from 7 to 19 mm in agar dilution studies^58^. Acetanisole was found to be antimicrobial^59^. Nonanal was found to be antimicrobial against *P. vulgaris* when isolated from the olive oil^60^. The major compounds of *Leptospermum* honeys with bioactivity against *C. albicans*, lacked activity against dermatophytes. A correlation was clearly observed between the presence of antifungal compounds in honey samples and the activity shown in the present study.

The major compounds detected in Jarrah honey were Isophorone (40.06%) and Nonanoic acid (5.62%). Isophorone is an antioxidant^19^, which has been detected by others in three other Australian Eucalyptus honeys as well as Sunflower honey^61^. In contrast, Spanish Eucalyptus honeys contained two major chemical marker compounds (2-hydroxy-5-methyl-3-hexanone and 3-hydroxy-5-methyl-2-hexanone), but Isophorone was absent^62^. In another study, Fir, Thyme and Orange blossom honeys from Greece were reported to have Isophorone in significant amounts. The compound was detected in Spanish Citrus, Rosemary, Eucalyptus, Lavender, Thyme and Heather honeys as reported by^63^. In addition, Alpha Isophorone was identified in Corsican strawberry-tree honey and defined as a chemical marker for that honey. Jarrah honey is derived from Jarrah tree which belongs to *Eucalyptus* species. Other countries such as Italy, Spain, Portugal and Israel also produce Eucalyptus honey, but these have different tastes and aromas due to different floral varieties and climate conditions, which affect volatile compound composition. Isophorone has been declared floral marker for Heather honey^64^ and Ulmo Chilean honey^13^. Another major compound observed in the present study was Nonanoic acid. This compound was also detected in other Australian Eucalyptus honeys^14^.

In summary, *Agastache* honey (40%) exhibited antifungal activity against dermatophytes and *C. albicans* in both the AWD and MD assays. The activity was fungicidal. Tea-tree showed fungicidal activity against *T. mentagrophytes* and *C. albicans*, but activity against *T. rubrum* was less than activity against the other fungal species tested. Manuka honey was active against *C. albicans* and showed some activity against dermatophytes. The activity was only fungistatic. Jelly bush, Super Manuka and Jarrah showed no activity against dermatophytes, but Jelly bush and Jarrah showed some activity against *C. albicans*. The volatile compound of all honeys were analysed by HS-SPME-GC-MS. This is the first time the volatile compounds of *Agastache* honey have been reported and screened for compounds possibly responsible for antifungal activity. *Agastache* honey contained several volatile compounds which have been reported to have antifungal activity whereas other honeys contained few antifungal compounds confirming the higher antifungal activity of *Agastache* honey than other honeys. The source of the volatile compounds were elucidated by detecting same compounds in both honey and flower. The study also identified chemical markers in each honey and identified common compounds in honey samples from different origin, for instance *Leptospermum* origin honeys contained Methyl 3,5-dimethoxybenzoate and Nonanal at variable percentages (Fig. 6). Based on higher abundance in each honey, the nominated chemical marker for honeys were: *Agastache*- Phenol-2, 4-bis (1, 1-dimethylethyl), Estragole and Nonanoic acid, ethyl ester; Manuka and Tea tree-ortho-Methoxyacetophenone, and Methyl 3,5-dimethoxybenzoate; Jelly bush-Linalool and Nonanal; Super Manuka-Methyl 3,5-dimethoxybenzoate and Nonanal; Jarrah honey- Isophorone and Nonanoic acid. The present findings that *Agastache* honey has greater antifungal activity than *Leptospermum* honeys, suggests that *Agastache* honey products could be developed for the topical application against skin-infections caused by fungi. However, this needs to be further confirmed by assessing honey *in-vivo* studies.

## Methods

### Sample preparation

Mono-floral *Agastache* honey was produced in a closed glass house as described by Anand *et al*.^3^ Other commercial honey samples: Manuka honey (hnz, UMF 22+, *Leptospermum scoparium*, produced in New Zealand), Tea-tree honey (Miellerie, *Leptospermum lanigerum* & *Leptospermum scoparium*, produced in Tasmania, Australia), Jelly bush honey (Australia’s Manuka, 20 + Active, *Leptospermum polygalifolium*, produced in New South Wales, Australia), Super Manuka honey (Berringa, MGO-400, *Leptospermum polygalifolium*, produced in Queensland, Australia) and Jarrah honey (Elixir, TA 45 + *Eucalyptus marginata*, produced in Perth, Australia) were purchased from a local store.

### Antifungal activity of honey

Two different methods were used to evaluate antifungal activity of honey: well-diffusion (dermatophytes), broth dilution (dermatophytes and *C. albicans*). The well-diffusion and micro-broth dilution methods were performed according to the guidelines of National Committee for Clinical Laboratories Standards^65^ and as described by the Alio S. *et al*.^66^.

### Inoculum size preparation

A clinical isolate of *C. albicans* (obtained from nail infection, Dorevitch pathology, Melbourne) and a reference strain of *C. albicans* (ATCC 10231) were maintained on Sabouraud Dextrose Agar (SDA, Oxoid, Australia) plates at 4 °C and sub-cultured prior to each experiment. *C. albicans* was suspended in sterile RPMI 1640 (Sigma Chemicals, St Louis, MO) and turbidity was adjusted to 0.5 McFarland, then suspensions were further diluted 1:100. *T. mentagrophytes* and *T. rubrum*, obtained from RMIT culture collection, were sub-cultured on SDA plates and incubated at 28 °C for 7 to 15 days. Both isolates sporulated well after this period. Stock conidial suspensions were prepared by covering the fungal colonies with 5 ml of sterile MilliQ water and gently rubbing with the tip of a sterile pipette tip. The suspensions were collected in sterile centrifuge tubes, counted using a haemocytometer. Conidial suspensions were adjusted to the desired density by adding RPMI 1640 medium with 10 g/l glucose (Sigma Chemicals, St Louis, MO) without sodium bicarbonate, to obtain a final concentration of 2 × 10^4^ to 6 × 10^4^ CFU/ml in 0.165 mol/l MOPS buffer (Gibco, Grand Island, NY), pH 7.0. Conidial concentration was verified by plating 10 ul of the adjusted conidial suspensions on SDA plates.

### Agar well diffusion assay

The agar well diffusion assay was used to assess the antifungal activity of *T. mentagrophytes* and *T. rubrum*. To perform the assay, SDA plates were flooded with 100 µl of RPMI media containing conidial suspensions (2 × 10^4^ to 6 × 10^4^ CFU/ml) of either *T. mentagrophytes* or *T. rubrum* and the suspensions were evenly spread. The plates were dried for 20 mins followed by punching four holes (10 mm) with a sterile cork borer at different sites. Diluted honey samples (80%, 40%, 20%, and 10% w/v) were placed in each well. Fluconazole was included as control. Fluconazole was dissolved in water and tested in the range of (128–0.05 ug/ml). Plates were allowed for pre-diffusion for 10–15 min before incubation at 28 °C for 7 days. The growth of dermatophytes was observed each day and diameters of zones of inhibition were recorded after 7 days.

### Broth micro-dilution assay

The minimal inhibitory concentration (MIC) and minimal fungicidal concentration (MFC) were determined for the honey samples against *C. albicans* and the two dermatophyte isolates according to the method described in Nature protocol^67^ with some modifications. The assays were performed in sterile, 96-well, flat-bottom, polystyrene microtiter plates (Corning Coster Ltd., NY). Briefly, *C. albicans* was streaked onto SDA plates and incubated for 24–48 hrs. Colony suspensions were prepared by touching 3–5 colonies with a sterile loop and transferring the inoculum into sterile 3–5 ml RPMI 1640. Turbidity was adjusted to 0.5 McFarland with sterile RPMI 1640, then suspensions were further diluted 1:100. Honey suspensions at variable concentrations (10%, 20%, 40% and 80%) were prepared in sterile RPMI, filtered through 0.45 µM filters and ten two-fold dilutions were made. Spore suspensions of the two dermatophytes were prepared as described in the previous section. 100 µl volumes of the diluted yeast or spore suspensions were inoculated into wells containing the honey dilutions resulting in the final inocula of ~5 × 10^5^ cfu/ml *C. albicans* or 2 × 10^3^ to 6 × 10^3^ cfu/ml dermatophyte suspension.

Sterility controls containing media only and growth controls containing yeast/dermatophytes only were included in the assay. Inoculum size was validated by removing 10 µl volumes from the growth control well and performing viable counts. To control for the colour of different honey samples, OD_600_ measurements were taken at 0 hrs and after 24–48 hrs incubation at 37 °C in the dark. Growth of yeast in the presence of honey was assessed by the following formula: OD_honey treated wells_/OD_negative control well_ × 100, where the control well was assigned 100% growth. To assess fungistatic activity, 10 µl samples from the first 2 wells containing no visible growth were plated onto the SDA plates and incubated for 24 hrs. The fungistatic end point was defined as the highest dilution showing growth inhibition. The fungicidal end point was defined as the highest dilution showing no growth on the inoculated plates. Since the hydrogen peroxide and glucose oxidase are light-sensitive, samples were incubated in the dark. All experiments were performed in triplicate.

### Hydrogen peroxide assay

Honey dilution initiates the production of hydrogen-peroxide since the glucose oxidase, an enzyme present in honey, is inactive in undiluted honey. Honeys used in this study were diluted to 40% and the production of hydrogen peroxide were measured as described in the protocol of OxiSelect™ Hydrogen Peroxide Assay Kit (Fluorometric) (catalogue number STA-344, Cell Biolabs, VIC, Australia). The H_2_O_2_ concentrations in the samples were calculated from the standard curve obtained by testing H_2_O_2_ in the concentration range of 0 µM – 200 µM. A fluorescence micro plate reader (Omega BMG LabTech, Australia) was used with an excitation of 544 nm and an emission of 590 nm. Experiments were performed three times in duplicate.

### HS-SPME/GC-MS analysis

#### Extraction of volatiles

The volatile compounds of honeys were determined according to the method described in Bianchi *et al*.^68^ with minor modifications. Briefly, honey samples (4 g) were dissolved in 2 ml of Milli Q water in a 10 ml vial hermetically capped with PTFE/silicon septum (Chromatographic Specialties Inc.). Diluted honey samples were equilibrated for 20 mins using a heating block and volatiles were extracted for 50 min at 60 °C. Samples were agitated by magnetic stirring to accelerate the transfer of analyte from the sample matrix to the coating fibre. An 85-µm polyacrylate (PA) fibre was fitted to the manual sampling fibre holder (Supelco, Bellefonte, PA). The precondition PA fibre (270 °C, 1 h) was inserted into the headspace of the vial containing the sample and vial was placed on the heating blocks. The extraction process was performed in triplicate.

#### Gas chromatography-mass spectrometry

The extracted volatiles were desorbed by placing the fibre into the gas chromatography injection port for 5 min. The identification of volatile compounds was performed as described by Yamani *et al*.^38^ using an Agilent 5973 MSD fitted with a DB-5 MS (5%-phenyl)-methylpolysiloxane fused silica column (Agilent) (30 m × 250 µm i.e. film thickness 0.25 µm). The analytical conditions were as follows; carrier gas helium (He 99.99%), flow rate 1.5 ml/min, split ratio 50:1. Initially, the oven temperature was 40 °C for 3 min, and it was raised later from 40 °C to 250 °C at 6 °C/min, where it was held for 5 min. The temperatures for injection port, transfer line and source temperatures were set at 250 °C, 280 °C, and 230 °C respectively. The samples were scanned in the mass range of 41–415 m/z as per Adams: Essential oil components by Quadrupole GC/MS^69^.

Spectra were acquired and processed using MSD ChemStation (E02.00.493). Compounds were identified with GC-MS reference libraries (Adams, Wiley 7th and NIST 2.0) using a 70% similarity match cut-off value. The peak area in the total ion chromatograms was the basis of calculations of concentration of studied compounds. The relative abundance was determined by integrating measurements obtained from three replicates.

### Statistical analysis

Minitab 18 (MiniTab Inc., PA, USA) was used to compare the fungal growth after treatment with different concentrations of honey. Analysis of variance (ANOVA) was followed by *post hoc* Tukey test and Kruskal-Wallis (KW) tests. Each experiment was conducted in triplicate and data were calculated from three different experiments. Significance was accepted as p ≤ 0.05.

## Supplementary information

Dataset S1-S6
