# Supplementary material for: Agastache honey has superior antifungal activity in comparison with important commercial honeys
Source: Sci Rep. 2019 Dec 3;9:18197. doi: 10.1038/s41598-019-54679-w (PMC6890684; doi:10.1038/s41598-019-54679-w)
Supplement: Supplementary file 1 — Dataset S1-S6 [file 41598_2019_54679_MOESM1_ESM.docx]

***Agastache* honey has superior antifungal activity in comparison with important commercial honeys**

**Sushil Anand^1*^, Margaret Deighton^1^, George Livanos^2^, Edwin Chi Kyong Pang^1^, and Nitin Mantri^1*^**

^1^ The Pangenomics Group, School of Science, RMIT University, Melbourne, 3083, Victoria, Australia

^2^ Kenkay Pharmaceuticals Pty Ltd., Smeaton Grange 2567, NSW, Australia

* Correspondence**:** A/Prof. Nitin Mantri [nitin.mantri@rmit.edu.au](mailto:nitin.mantri@rmit.edu.au) or Sushil Anand sush.anand@gmail.com

**Supplementary Tables S1-S6**

**Table S1.** Volatile compounds identified in *Agastache* honey using HS-SPME-GC-MS

| **No.** | **PtR** | **Area %** | **Compound (Adams KI)** |
| --- | --- | --- | --- |
| 1 | 10.77 | 2.56 | Benzaldehyde |
| 2 | 12.64 | 0.38 | D-Limonene |
| 3 | 13.03 | 1.11 | Benzeneacetaldehyde |
| 4 | 14.62 | 3.19 | Nonanal |
| 5 | 14.85 | 1.73 | Phenylethyl Alcohol |
| 6 | 15.07 | 2.14 | 1H-Pyrazole, 4,5-dihydro-5,5-dimethyl-4-isopropylidene- |
| 7 | 15.36 | 0.23 | Cyclopentasiloxane, decamethyl- |
| 8 | 15.66 | 0.97 | 2,6,6-Trimethyl-2-cyclohexene-1,4-dione (4 Ketoisophorone) |
| 9 | 16.90 | 1.36 | Octanoic acid, ethyl ester (ethyl octnoate) |
| 10 | 17.00 | 12.31 | Estragole |
| 11 | 17.16 | 2.42 | Decanal |
| 12 | 18.36 | 5.17 | Benzaldehyde, 4-methoxy- (p-anisaldehyde) |
| 13 | 19.22 | 7.22 | Nonanoic acid, ethyl ester |
| 14 | 19.38 | 2.54 | Benzene, 1-methoxy-4-propyl- |
| 15 | 19.69 | 2.76 | Phenol, 2,3,5-trimethyl- |
| 16 | 20.80 | 1.19 | Nonanoic acid |
| 17 | 20.99 | 1.63 | Propanoic acid, 2-methyl-, 2-ethyl-3-hydroxyhexyl ester |
| 18 | 21.63 | 2.34 | 2H-Benzimidazol-2-one, 1,3-dihydro-5-methyl- |
| 19 | 22.11 | 4.67 | Bicyclo undec-4-ene, 4,11,11-trimethyl-8-methylene (β-Caryophyllene) |
| 20 | 22.63 | 3.53 | Benzoic acid, 4-methoxy-, ethyl ester |
| 21 | 23.01 | 6.32 | 2-Propenoic acid, 3-phenyl-, ethyl ester |
| 22 | 23.51 | 0.85 | Naphthalene, 1,2,3,5,6,7,8,8a-octahydro-1,8a-dimethyl-7-1-methylethenyl (Y-cadinene) |
| 23 | 23.75 | 12.77 | Phenol, 2,4-bis(1,1-dimethylethyl) |
| 24 | 25.16 | 1.14 | Benzoic acid, 3,5-dimethoxy-, methyl ester |
| 25 | 25.47 | 2.96 | Dodecanoic acid, ethyl ester |
| 26 | 26.33 | 1.87 | 2-Naphthalenemethanol, 1,2,3,4,4a,5,6,7-octahydro-.alpha.,.alpha.,4a,8-tetramethyl-, (2R-cis) |
| 27 | 27.49 | 0.82 | Heptadecane |
| 28 | 30.40 | 0.41 | Homosalate |
| 29 | 30.97 | 2.90 | Nonadecane |
| 30 | 32.46 | 5.68 | Hexadecanoic acid, ethyl ester |
| 31 | 34.14 | 2.32 | Heneicosane |
| 32 | 34.83 | 2.48 | 9,12-Octadecadienoic acid (Z,Z)- |

PtR - Predicted retention time

**Table S2.** Volatile compounds identified in Manuka honey using HS-SPME-GC-MS

| **No.** | **PtR** | **Area %** | **Compound (Adams KI)** |
| --- | --- | --- | --- |
| 1 | 6.19 | 0.09 | Octane |
| 2 | 7.00 | 0.21 | Furfural |
| 3 | 9.44 | 0.07 | Anisole |
| 4 | 10.76 | 1.47 | Benzaldehyde |
| 5 | 11.70 | 0.27 | Benzofuran |
| 6 | 11.92 | 0.11 | Octanal |
| 7 | 12.17 | 0.31 | 2-Acetyl-5-methylfuran |
| 8 | 13.03 | 1.48 | Benzeneacetaldehyde |
| 9 | 13.60 | 0.53 | Acetophenone |
| 10 | 14.50 | 0.39 | Linalool |
| 11 | 14.63 | 3.87 | Nonanal |
| 12 | 14.87 | 0.25 | Phenylethyl Alcohol |
| 13 | 15.19 | 0.08 | Benzyl methyl ketone |
| 14 | 15.36 | 4.34 | Benzene, 1-ethenyl-4-methoxy- |
| 15 | 16.06 | 1.29 | Ethanone, 1-(2-hydroxyphenyl)- |
| 16 | 16.87 | 0.29 | Methyl salicylate |
| 17 | 17.16 | 0.45 | Decanal |
| 18 | 17.50 | 0.13 | 3-Cyclohexene-1-acetaldehyde, .alpha.,4-dimethyl- |
| 19 | 17.96 | 0.13 | Benzaldehyde, 2-methoxy- |
| 20 | 18.37 | 0.38 | Benzaldehyde, 4-methoxy- |
| 21 | 18.60 | 0.13 | Bicyclo[3.1.1]heptane-2-methanol, 6,6-dimethyl- |
| 22 | 18.80 | 1.53 | Nonanoic acid |
| 23 | 19.22 | 46.9 | Acetanisole (ortho-Methoxyacetophenone) |
| 24 | 20.28 | 0.77 | 3-cyano-4-isopropenyl-5-methylpyridine |
| 25 | 20.40 | 0.17 | 2-methyl-5-(phenylmethyl)furan |
| 26 | 20.55 | 1.43 | Benzene, 2,4-diisocyanato-1-methyl- |
| 27 | 20.83 | 0.80 | n-Decanoic acid |
| 28 | 20.92 | 0.28 | Benzoic acid, 2-methoxy-, phenyl ester |
| 29 | 21.49 | 1.07 | Ethanone, 1-(2-hydroxy-6-methoxyphenyl)- |
| 30 | 21.65 | 0.22 | 2H-Benzimidazol-2-one, 1,3-dihydro-5-methyl- |
| 31 | 22.17 | 0.41 | Naphthalene, 2,6-dimethyl- |
| 32 | 22.80 | 0.19 | Naphthalene, 1,3-dimethyl- |
| 33 | 23.01 | 0.15 | 2-Propenoic acid, 3-phenyl-, ethyl ester |
| 34 | 23.71 | 0.17 | 2-Cyclohexen-1-one, 3-(2-butenyl)-2,4,4-trimethyl- |
| 35 | 24.32 | 0.07 | Benzenamine, N,N-diethyl-3-methyl- |
| 36 | 24.71 | 0.22 | Naphthalene, 1,2,3,4-tetrahydro-5,7-dimethyl- |
| 37 | 25.20 | 26.67 | Benzoic acid, 3,5-dimethoxy-, methyl ester |
| 38 | 25.61 | 0.08 | Benzene, 1,3,5-tris(1-methylethyl)- |
| 39 | 26.25 | 0.51 | Benzophenone |
| 40 | 26.33 | 0.26 | gama.-eudesmol |
| 41 | 28.04 | 0.44 | 4H-Pyrido[1,2-a]pyrimidine-3-carboxylic acid, 1,6,7,8,9,9a-hexahydro-4-oxo-, ethyl ester |
| 42 | 29.36 | 0.73 | 2-Ethylhexyl salicylate |
| 43 | 30.27 | 0.07 | 1,2-Benzenedicarboxylic acid, bis(2-methylpropyl) ester |
| 44 | 32.11 | 0.01 | 6-(N-Allylamino)-7-methylquinoline-5,8-dione |
| 45 | 34.14 | 0.25 | Heneicosane |
| 46 | 37.05 | 0.09 | Eicosane |

PtR - Predicted retention time

**Table S3.** Volatile compounds identified in Tea-tree honey using HS-SPME-GC-MS

| **No.** | **PtR** | **Area %** | **Compound (Adams KI)** |
| --- | --- | --- | --- |
| 1 | 5.88 | 0.47 | Octane |
| 2 | 10.45 | 0.74 | Benzaldehyde |
| 3 | 11.37 | 0.07 | Benzofuran |
| 4 | 12.18 | 0.08 | Cymene <Ortho> |
| 5 | 12.29 | 0.55 | 1-Hexanol, 2-ethyl- |
| 6 | 12.69 | 7.70 | Benzene acetaldehyde |
| 7 | 13.29 | 0.44 | Acetophenone |
| 8 | 14.18 | 4.51 | Linalool |
| 9 | 14.29 | 8.25 | Nonanal |
| 10 | 15.37 | 2.82 | 2-Hydroxy-3,5,5-trimethyl-cyclohex-2-enone |
| 11 | 15.72 | 0.47 | Ethanone, 1-(2-hydroxyphenyl)- |
| 12 | 15.98 | 4.51 | Nonanol <N-> |
| 13 | 16.53 | 0.25 | Methyl Salicylate |
| 14 | 16.82 | 1.09 | Decanal |
| 15 | 18.47 | 6.12 | Nonanoic acid |
| 16 | 19.09 | 31.39 | Acetanisole <Ortho-> (Methoxyacetophenone) |
| 17 | 19.92 | 0.32 | 1-Naphthalenol, 4-methyl- |
| 18 | 20.08 | 0.38 | Benzene, 1,3-diisocyanato-2-methyl- |
| 19 | 20.21 | 8.31 | Benzene, 2,4-diisocyanato-1-methyl- |
| 20 | 21.41 | 2.10 | 2H-Benzimidazol-2-one, 1,3-dihydro-5-methyl- |
| 21 | 23.42 | 1.11 | Phenol, 2,4-bis(1,1-dimethylethyl)- |
| 22 | 23.80 | 0.14 | Calamenene <Cis> |
| 23 | 24.80 | 14.47 | Benzoic acid, 3,5-dimethoxy-, methyl ester |
| 24 | 25.88 | 0.42 | Benzophenone |
| 25 | 27.14 | 0.69 | Heptadecane |
| 26 | 30.60 | 2.48 | Nonadecane |

PtR - Predicted retention time

**Table S4.** Volatile compounds identified in Jelly Bush honey using HS-SPME-GC-MS

| **No.** | **PtR** | **Area %** | **Compound (Adams KI)** |
| --- | --- | --- | --- |
| 1 | 3.32 | 0.12 | Acetic acid |
| 2 | 4.80 | 0.12 | Disulfide, dimethyl |
| 3 | 6.17 | 0.33 | Octane |
| 4 | 7.03 | 1.86 | Furfural |
| 5 | 9.43 | 0.09 | Anisole |
| 6 | 10.75 | 6.75 | Benzaldehyde |
| 7 | 11.91 | 0.28 | Octanal |
| 8 | 12.16 | 0.69 | 2-Acetyl-5-methylfuran |
| 9 | 12.64 | 1.65 | D-Limonene |
| 10 | 13.02 | 2.25 | Benzene acetaldehyde |
| 11 | 13.42 | 0.11 | .gamma.-Terpinene |
| 12 | 13.77 | 4.34 | p-Cresol |
| 13 | 14.52 | 19.44 | 1,6-Octadien-3-ol, 3,7-dimethyl- |
| 14 | 14.64 | 12.49 | Nonanal |
| 15 | 14.84 | 1.01 | Phenylethyl Alcohol |
| 16 | 15.77 | 1.00 | Lilac aldehyde C |
| 17 | 15.82 | 0.75 | Neroloxide |
| 18 | 16.95 | 9.16 | alpha.-Terpineol |
| 19 | 17.15 | 0.98 | Decanal |
| 20 | 17.34 | 0.30 | Naphthalene, 1,2,3,4-tetrahydro-1,1,6-trimethyl- |
| 21 | 17.49 | 0.84 | 3-Cyclohexene-1-acetaldehyde, .alpha.,4-dimethyl- |
| 22 | 17.54 | 0.69 | 1,3-Cycloheptadiene |
| 23 | 17.59 | 0.69 | Furan, 3-phenyl- |
| 24 | 18.01 | 0.65 | Benzene acetic acid, ethyl ester |
| 25 | 18.94 | 4.33 | Nonanoic acid |
| 26 | 19.07 | 2.93 | Ortho-Methoxy acetophenone |
| 27 | 19.68 | 7.82 | Phenol, 3,4,5-trimethyl- |
| 28 | 21.52 | 0.40 | Benzoic acid, 2-methoxy-, ethyl ester |
| 29 | 21.65 | 0.23 | 2H-Benzimidazol-2-one, 1,3-dihydro-5-methyl- |
| 30 | 23.75 | 1.04 | Phenol, 2,4-bis(1,1-dimethylethyl)- |
| 31 | 24.57 | 0.14 | alpha.-Calacorene |
| 32 | 25.18 | 11.70 | Benzoic acid, 3,5-dimethoxy-, methyl ester |
| 33 | 26.25 | 0.32 | Benzophenone |
| 34 | 28.64 | 2.82 | Benzoic acid, 4-hydroxy-3,5-dimethoxy-, hydrazide |
| 35 | 29.35 | 0.50 | 2-Ethylhexyl salicylate |
| 36 | 30.27 | 0.11 | 1,2-Benzenedicarboxylic acid, bis(2-methylpropyl) ester |
| 37 | 30.97 | 0.76 | Nonadecane |
| 38 | 32.46 | 0.14 | Hexadecanoic acid, ethyl ester |

PtR - Predicted retention time

**Table S5.** Volatile compounds identified in Super Manuka honey using HS-SPME-GC-MS

| **No.** | **PtR** | **Area %** | **Compound (Adams KI)** |
| --- | --- | --- | --- |
| 1 | 5.89 | 0.56 | Octane |
| 2 | 6.80 | 1.23 | Furfural |
| 3 | 9.14 | 0.17 | Anisole |
| 4 | 9.64 | 0.42 | Pinene <Alpha-> |
| 5 | 10.44 | 5.13 | Benzaldehyde |
| 6 | 10.63 | 0.21 | Trisulfide, dimethyl |
| 7 | 11.24 | 0.12 | Linalool Oxide<Dehydroxy-Trans-> |
| 8 | 11.60 | 0.64 | Octanal |
| 9 | 12.19 | 0.30 | Cymene<Ortho-> |
| 10 | 12.32 | 0.36 | Limonene |
| 11 | 12.58 | 0.41 | Benzyl alcohol |
| 12 | 12.71 | 1.95 | Benzene acetaldehyde |
| 13 | 13.31 | 0.16 | Acetophenone |
| 14 | 13.44 | 1.91 | Linalool oxoid<Cis->(Furanoid) |
| 15 | 13.61 | 0.48 | Benzene, 1-methyl-4-(1-methylethyl) (p-Cymene ) |
| 16 | 14.19 | 2.12 | Linalool |
| 17 | 14.31 | 11.31 | Nonanal |
| 18 | 15.07 | 0.14 | Cyclopentasiloxane, decamethyl |
| 19 | 15.38 | 0.27 | 2-Hydroxy-3,5,5-Trimethyl-2-Cyclohexenonone |
| 20 | 16.54 | 0.43 | Methyl salicylate |
| 21 | 16.62 | 1.22 | Sylvestrene <Iso-> |
| 22 | 16.66 | 2.95 | Anethole<Z-> |
| 23 | 16.83 | 1.09 | Decanal |
| 24 | 17.20 | 0.55 | 3-(CYCLOHEX-3'-EN-YL)PROPIONALDEHYDE |
| 25 | 18.38 | 4.53 | Phenol, 2,3,5-trimethyl- |
| 26 | 19.40 | 8.64 | Phenol, 3,4,5-trimethyl- |
| 27 | 20.08 | 0.86 | Benzene, 1,3-diisocyanato-2-methyl- |
| 28 | 20.22 | 7.68 | Benzene, 2,4-diisocyanato-1-methyl- |
| 29 | 21.06 | 0.33 | Tetradecene<1-> |
| 30 | 21.75 | 0.33 | Caryophyllene<E-> |
| 31 | 23.31 | 0.15 | Pentadecane |
| 32 | 23.43 | 1.51 | Phenol, 2,4-bis(1,1-dimethylethyl)- |
| 33 | 24.84 | 40.09 | Benzoic acid, 3,5-dimethoxy-, methyl ester |
| 34 | 25.13 | 0.41 | Hexadecanol <N-> |
| 35 | 25.89 | 0.38 | Benzophenone |
| 36 | 27.14 | 0.35 | Heptadecane |
| 37 | 28.99 | 0.23 | Salicylcate (2-ethylhexyl) |
| 38 | 33.77 | 0.42 | Heneicosane |

PtR - Predicted retention time

**Table S6.** Volatile compounds identified in Jarrah honey using HS-SPME-GC-MS

| **No.** | **PtR** | **Area %** | **Compound (Adams KI)** |
| --- | --- | --- | --- |
| 1 | 1.97 | 0.27 | Methane, thiobis- |
| 2 | 2.00 | 0.20 | Methane, thiobis- |
| 3 | 2.03 | 0.34 | Methane, thiobis- |
| 4 | 4.10 | 0.23 | 2-Butanone, 3-hydroxy- |
| 5 | 6.92 | 1.71 | Acetyl valeryl |
| 6 | 7.36 | 0.33 | (Z)-2-(Aminomethylene)-3,3-dimethylbutanenitrile |
| 7 | 8.75 | 0.21 | 4-Methyl-2-hexanol |
| 8 | 10.48 | 0.29 | Benzaldehyde |
| 9 | 12.19 | 0.87 | Cymene<Ortho> |
| 10 | 12.64 | 0.19 | 3-Cyclohexen-1-one, 3,5,5-trimethyl- |
| 11 | 12.71 | 1.16 | Benzeneacetaldehyde |
| 12 | 13.95 | 0.61 | Cymenene<Para-> |
| 13 | 14.19 | 0.81 | Linalool |
| 14 | 14.31 | 2.58 | Nonanal |
| 15 | 14.77 | 40.06 | Isophorone |
| 16 | 15.07 | 0.25 | Cyclopentasiloxane, decamethyl- |
| 17 | 15.25 | 0.28 | Cyclohexanol, 4-(1-methylethyl)- |
| 18 | 15.48 | 0.67 | Ethanone, 1-(1,4-dimethyl-3-cyclohexen-1-yl)- |
| 19 | 15.71 | 5.12 | 2-Hydroxy-3,5,5-Trimethyl-2-Cyclohexenone |
| 20 | 16.18 | 1.26 | Octanoic Acid |
| 21 | 16.62 | 1.35 | Terpineol<alpha-> |
| 22 | 16.72 | 1.12 | Benzenemethanol, .alpha.,.alpha.,4-trimethyl- |
| 23 | 16.70 | 0.95 | 1,3-Cyclohexadiene-1-carboxaldehyde, 2,6,6-trimethyl- |
| 24 | 16.83 | 1.29 | Decanal |
| 25 | 17.27 | 1.42 | Furan, 3-phenyl- |
| 26 | 17.75 | 0.82 | Cumin aldehyde |
| 27 | 18.45 | 5.62 | Nonanoic acid |
| 28 | 18.70 | 0.55 | Cymen-7-ol<Para-> |
| 29 | 18.89 | 2.18 | Thymol |
| 30 | 19.04 | 0.99 | Phenol, 2-methyl-5-(1-methylethyl)- |
| 31 | 20.22 | 3.75 | Benzene, 2,4-diisocyanato-1-methyl- |
| 32 | 20.88 | 2.22 | Decanoic acid |
| 33 | 21.65 | 0.32 | 2-Propenoic acid, 3-phenyl- |
| 34 | 21.42 | 2.81 | 2H-Benzimidazol-2-one, 1,3-dihydro-5-methyl- |
| 35 | 22.93 | 2.84 | 9,9-dimethyl-9,-10-dihydroanthacene |
| 36 | 23.31 | 1.71 | Pentadecane |
| 37 | 24.17 | 0.29 | Coumarin, 3,4-dihydro-4,4,7-trimethyl- |
| 38 | 24.27 | 3.44 | (+-)-(5,6,7,8-Tetrahydro-4-methyl-1-naphthalenyl)-1-ethanone |
| 39 | 24.82 | 6.69 | Benzoic acid, 3,5-dimethoxy-, methyl ester |
| 40 | 25.89 | 0.81 | Benzophenone |
| 41 | 26.80 | 0.61 | 2-Cyclohexen-1-one, 3,5,5-trimethyl-4-(3-oxobutyl)- |
| 42 | 27.90 | 0.37 | 1,2-Benzenedicarboxylic acid, bis(2-methylpropyl) ester |
| 43 | 30.41 | 0.48 | Homo menthyl salicylate |

PtR - Predicted retention time
